# Protein expression profiling during chick retinal maturation: a proteomics-based approach

**DOI:** 10.1186/1477-5956-6-34

**Published:** 2008-12-10

**Authors:** Sorcha Finnegan, Joanne L Robson, Mildred Wylie, Adrienne Healy, Alan W Stitt, William J Curry

**Affiliations:** 1 Centre for Vision Sciences, Queen's University of Belfast, Institute of Clinical Sciences, Grosvenor Road, Royal Victoria Hospital, Belfast, BT12 6BA, Northern Ireland; 2 School of Biological Sciences, Queen's University of Belfast, Medical Biology Centre, 97 Lisburn Road, Belfast, BT9 7BL, Northern Ireland; 3 School of Biological and Biomedical Sciences, Durham University, South Road, Durham, DH1 3LE, UK; 4 Veterinary Sciences Division, Department of Agriculture and Rural Development, Stoney Road, Stormont, Belfast BT4 3SD, Northern Ireland

## Abstract

**Background:**

The underlying pathways that drive retinal neurogenesis and synaptogenesis are still relatively poorly understood. Protein expression analysis can provide direct insight into these complex developmental processes. The aim of this study was therefore to employ proteomic analysis to study the developing chick retina throughout embryonic (E) development commencing at day 12 through 13, 17, 19 and post-hatch (P) 1 and 33 days.

**Results:**

2D proteomic and mass spectrometric analysis detected an average of 1514 spots per gel with 15 spots demonstrating either modulation or constitutive expression identified via MS. Proteins identified included alpha and beta-tubulin, alpha enolase, B-creatine kinase, gamma-actin, platelet-activating factor (PAF), PREDICTED: similar to TGF-beta interacting protein 1, capping protein (actin filament muscle Z line), nucleophosmin 1 (NPM1), dimethylarginine dimethylaminohydrolase, triosphoaphate isomerase, DJ1, stathmin, fatty acid binding protein 7 (FABP7/B-FABP), beta-synuclein and enhancer of rudimentary homologue.

**Conclusion:**

This study builds upon previous proteomic investigations of retinal development and represents the addition of a unique data set to those previously reported. Based on reported bioactivity some of the identified proteins are most likely to be important to normal retinal development in the chick. Continued analysis of the dynamic protein populations present at the early stages and throughout retinal development will increase our understanding of the molecular events underpinning retinogenesis.

## Background

Retinogenesis is an intricate process in which pluripotent progenitor cells resident in a pseudostratified neuroepithelium differentiate to form the mature stratified retina composed of specialized neural and glial cells [1]; it has been studied in diverse species such as zebrafish,*Drosophila*, mouse and chick [2-5]. In the chick retina neurogenesis commences at E2 in a dorsotemporal area of the central primordial retina and spreads towards the periphery until E13 [6]. It proceeds in a well-defined chronological sequence; in the dorsal retina ganglion cells are generated first (between E2–E8), followed by overlapping phases of amacrine (E3–E9), horizontal (E4–E8), photoreceptor (E4–E8), Müller (E4–E11) and bipolar cell production (E6–E13) [6]. The inner plexiform layer (IPL) is observable by E7–E8 and the outer plexiform layer (OPL) is evident by E9 and synaptogenesis is apparent at E13 in the IPL [7] and at E16 in the OPL [7,8]. The characteristic anatomically layered retina is evident by E19 and at P 1 the maturing photoreceptor outer segments are obvious [6].

The chick visual system has long been recognized as one of the most valuable tools to study neural development, and, more recently retinal pathologies. An advantageous feature of the chick eye is the experimental accessibility from the earliest stages of development; the eye is relatively large at early embryonic stages, with compartmentalisation of the visual system evident after about 30 hours of incubation and ocular development mostly completed by the time of hatching [9]. Recently the chicken genome has been completed, there are one billion base pairs of DNA (about 1/3 as many as humans) and 20,000 – 30,000 genes [10]; the conservation of gene order between the chicken and human genomes is similar to that between humans and mice, in spite of the much greater evolutionary divergence [11] and it is therefore possible to predict both candidate disease loci and candidate genes by comparison with the human genome [12].

Proteomic methodologies have been employed to study ontogeny; development of the cerebella [13], brain [14], lung [15] and whole embryonic development [16]. Retinal proteome studies to date have focused on the analysis of adult tissues from diverse species [17-22]. Three studies to date have employed the chick to investigate aspects of the developing retinal proteome [21,23,24].

This study aimed to characterise the developing chick retinal proteome focusing on key areas during retinal development, neurogenesis and synaptogenesis and with retinal maturation. This information about holistic protein expression during retinogenesis is fundamental for directing retinal stem cell research which may in turn better enable the aspiration of applying stem cell therapies to halt, repair or replace degenerative retinal cells especially in pathologies such as retinitis pigmentosa (RP) for which there are yet no real treatment options and no cures [25].

## Materials and methods

### Retinal Tissue Collection

Fertilized White Leghorn chicken (*Gallus gallus domesticus*) eggs were incubated in a humidified atmosphere (56% CO_2_) at 38°C and embryos (E5–E20) where killed by chilling in -80°C for 15 minutes. P1 chicks were killed by cervical dislocation. Eyes were ressected, retinae removed (within 2 hours of death), snap frozen in liquid nitrogen and stored at -80°C. All animal procedures were performed in compliance with the UK Animals (Scientific Procedures) Act 1986.

The heads from White Leghorn chicken (*Gallus gallus domesticus*) (age 33 days) slaughtered by electrocution and decapitation for food production (Moy Park Ltd, Dungannon, Northern Ireland) were transported to the laboratory on ice. Eyes were ressected, retina gently removed, snap-frozen, homogenised in liquid nitrogen and stored at -80°C. Retinal tissues were recovered as described within 3 hours of slaughter.

### 2D PAGE

In brief, retinal tissues were ground to a powder in liquid nitrogen and dithiothreitol (DTT) (3.1 mg) (Melford Labs, UK) was added to 1 ml of 40 mM ammonium bicarbonate extraction buffer (approx 5:1 (w/v)), sonicated (15 min, 4°C), extracted for 1 hr on ice with vortexing, dialysed (24 hr, 4°C) using Slide-A-Lyzer^® ^dialysis cassettes, 3500 MWCO (Pierce, USA), lyophilized and reconstituted in lysis solution (7 M urea, 2 M thiourea, 2% CHAPS, 20 mM DTT, 0.5% IPG buffer pH 4–7) and protein quantification was performed (PlusOne 2-D Quant Kit, Amersham Biosciences, UK). Retinal protein extract (1 mg) was diluted to generate a final volume of 400 μl with rehydration buffer (7 M urea, 2 M thiourea, 2% CHAPS, 80 mM DTT, 0.5% IPG buffer containing 3', 3", 5', 5" tetrabromophenolsulfonephthalein (bromophenol blue) (Sigma-Aldrich, UK) and employed to rehydrate 18 cm pH 4–7 IPG strips (Amersham Biosciences, UK).

Following rehydration (18 hr, room temp), IEF was performed using a Multiphor II system (20°C) with a MultiTemp III circulator and an EPS 3501 XL power supply (200 V for 1 Volt hour (Vh), 3500 V for 3000 Vh and held at 35000 for 52000 Vh) (Amersham Biosciences, UK).

IPG strips were equilibrated in 10 ml equilibration buffer (50 mM Tris-Cl, pH 8.8, 6 M urea, 30% (v/v) glycerol, 2% (w/v) SDS, bromophenol blue) with 100 mg DTT (15 min) and, in 10 ml of fresh equilibration buffer containing 250 mg iodoacetamide (15 min) (Sigma-Aldrich, UK). SDS-PAGE was performed using an Ettan™ DALT *six *vertical system (Amersham Biosciences, UK) with a MultiTemp™ III Thermostatic Circulator (25°C) (Amersham Biosciences, UK) using 600 V, 400 mA for 30 minutes and at 600 V, 400 mA, 17 W/gel for 4 hours. (Two retina from one chick were used for 1 gel and 3 different chicks were used at each age to produce 3 × 2D gels i.e. for each gel at each age, each gel is representative of one animal. Triplicate gels were run at the same time for each age in order to limit any differences in experimental conditions). Gels were fixed, stained with Coomassie Brilliant Blue G-colloidal (Sigma-Aldrich, UK) for 24 hr and destained using standard procedures and stored in 25% (v/v) ammonium sulphate (BDH Laboratories, UK) solution at 4°C.

### Gel image analysis

Gels were scanned as 16 bit gray scale Tif image files with a GS-800 calibrated densitometer (Bio-Rad Laboratories Inc., Hercules, USA) and image analysis was performed using Progenesis PG220 (Nonlinear Dynamics Ltd., Newcastle upon Tyne, UK). Gels were imported and spot detection and matching was performed automatically in Progenesis followed by manual adjustment. Briefly, spots were detected (minimum spot radius of 16, minimum spot intensity and split factor 7) and volumes were normalized (using the total spot volume method, meaning all spot volumes are expressed as a fraction of the total spot volume of any given gel. This allows correction for potential differences in protein loading between gels. When this has been performed the measurements for any given spot in replicate gels will have adjusted to the same level, meaning that differences in protein loading have been compensated for, therefore any differences detected in protein expression are due to biological changes) throughout the entire set of gels within the experiment. Average gels were then created by the software to allow spot pattern comparison. These gels are a statistical combination of all gels in a group showing average spot values with associated error. For this study an average gel was created for each age by combining the three gels at each age. The criteria for including a spot in an average gel were that the spot must be present in at least two of the three individual gels.

In order to begin to analyse protein expression changes between ages the average gel representing E12 was compared to average gel representing P33 simply for the purposes of assessing significant changes in protein expression between these two ages; the following parameters were used for this; only those filtered spots exceeding an intensity threshold of 1.5 to 2 fold increase or decrease between the two ages were subjected to further analysis, the threshold regulation factor for the significance level was set at p ≤ 0.05; spots regulated more than the factor required for significance were further considered as candidate spots and subsequently subjected to manual verification. After this the spots that showed significant difference in expression between E12 and P33 were investigated at all other ages in order to map expression changes over time. Spots displaying significant expression changes were subsequently identified by MS.

### Mass Spectrometry

Proteins analysed by MALDI-TOF (Durham University, UK) were tryptically digested using a Genomic Solutions ProGest (Genomic Solutions^®^, USA), transferred to a micro-titre plate (PE Biosystems Symbiot robot), lyophilised in a vacuum concentrator, resuspended in 10 ul of 0.1% formic acid, sonicated and spotted onto a MALDI target plate prior to MS analysis using a Voyager DE-STR MALDI-TOF mass spectrometer (PE Biosystems, USA). Peak lists were generated with the MASCOT Wizard and submitted to Mascot (version 2.1) for peptide mass fingerprint database searching of the National Center for Biotechnology Information (NCBI) database. The following search parameters were used; Type of search: Peptide Mass Fingerprint, Enzyme: Trypsin, Fixed modifications: Carbamidomethyl (C), Variable modifications: Oxidation (M), Mass values: Monoisotopic, Protein Mass: Unrestricted, Peptide Mass Tolerance: ± 50 ppm, Peptide Charge State: 1+, Max Missed Cleavages: 1.

Additional MS was completed in Queen's University Belfast using the following preparative protocols. Protein spots of interest were excised, the gel piece was transferred to siliconised Eppendorf tubes, washed repeatedly with vortexing in 100 μl water until a neutral pH was achieved. Destaining solution (100 μl, 50 mM ammonium bicarbonate/40% (v/v) ethanol, pH 7.8) was added with vortexing until the Coomassie stain was not evident. The gel was macerated, incubated with 25 μl of acetonitrile (15 min) and repeated until the gel was dehydrated, finally acetonitrile was removed under vacuum (SpeedVac, Savant, UK). The sample was incubated (37°C, 18 hr) with sequencing grade modified trypsin (Promega, Southampton, UK) (20 μl/20 μg) dissolved in 25 mM ammonium bicarbonate. Subsequently, 30 μl of 50% (v/v) acetonitrile containing 5% (v/v) TFA was added with vortexing and sonication (Bandelin Sonorex RK 31, Berlin) for 6 min followed by centrifugation to pellet the gel. The supernatant was decanted and dried under vacuum. The dried digests were stored at -80°C until MS analysis.

### nanoESI-MS/MS

Prior to ESI-MS/MS, samples were desalted using C_18 _ZipTip^® ^(Millipore, UK) pipette tips as per the manufacturer's instructions. Using a GELoader^® ^Tip (Eppendorf AG, Hamburg, Germany), each sample was loaded into a palladium-coated, borosilicate glass nano-electrospray capillary (Proxeon Biosystems, Odense, Denmark) prior to mass spectrometry using a ThermoFinnigan LCQ Deca mass spectrometer (ThermoFinnigan, San Jose, U.S.A.) with a capillary voltage of 36 V, 190°C and collision energy was 35% with helium as the collision gas. Data generated by nanoESI-MS/MS was analysed as follows: Xcalibar binary (RAW) files were converted into peak list (DTA) files for each precursor mass. DTA files were merged into a single file, submitted to the MASCOT search engine [26,27]; An MS/MS ion search was performed and Mascot was set to query the National Center for Biotechnology Information (NCBI) database. The following search parameters were used: monoisotopic molecular masses, trypsin specificity, all species allowed, one missed cleavage site, and peptide charge = 1+. The peptide mass and MS/MS mass tolerances were set at 1.2 Da and 0.6 Da, respectively.

### Histology

Resected eyes were perforated and immersion fixed in buffed 4% (w/v) paraformaldehyde (4 hr, 4°C) (Sigma-Aldrich, UK), dehydrated in a series of alcohols and xylene to render the tissue miscible with wax impregnation, embedding and microtome sectioning (5 μm).

## Results

### Retinal development in the chick

Routine histological analysis was employed to verify previous reported patterns of chick retinal development [1,9,28] (data not shown).

### Proteomic analysis of the developing retina

**Figure 1:**
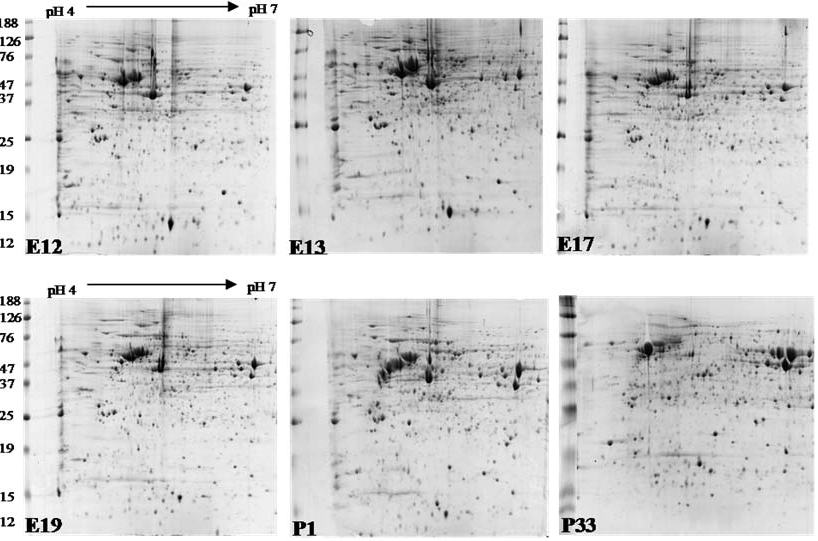
**Representative 2D protein profiles from embryonic and post hatch chick retina**. Retinal protein was extracted in 40 mM ammonium bicarbonate and 1 mg of extracted protein was separated in the first dimension between pH 4 to 7 on an 18 cm IPG strip, and, in the second dimension on a 12% polyacrylamide gel (25 cm × 20 cm). Proteins were visualised with Coomassie blue.

Embryonic and post hatch chick retinal extracts were focused using IPG pH 4 – 7 and arrayed using 12% polyacrylamide gels in the second dimension (Fig 1). Analysis of the average number of spots observed on triplicate 2D gels (each representing one sample, *n = 3*) detected no significant difference between the numbers of spots detected (Table 1).

**Table 1:** Number of Spots detected on 2D gels by Progenesis image analysis Software

	**E12**	**E13**	**E17**	**E19**	**P1**	**P33**
**Gel 1**	1525	1283	1506	1602	1457	1704

**Gel 2**	1412	1594	1533	1530	1695	1678

**Gel 3**	1558	1498	1510	1510	1576	1082

**Average**	1498	1555	1516	1547	1576	1704

**Standard Error of the mean**	44.2	33.3	8.4	28	68.7	9.3

**Figure 2:**
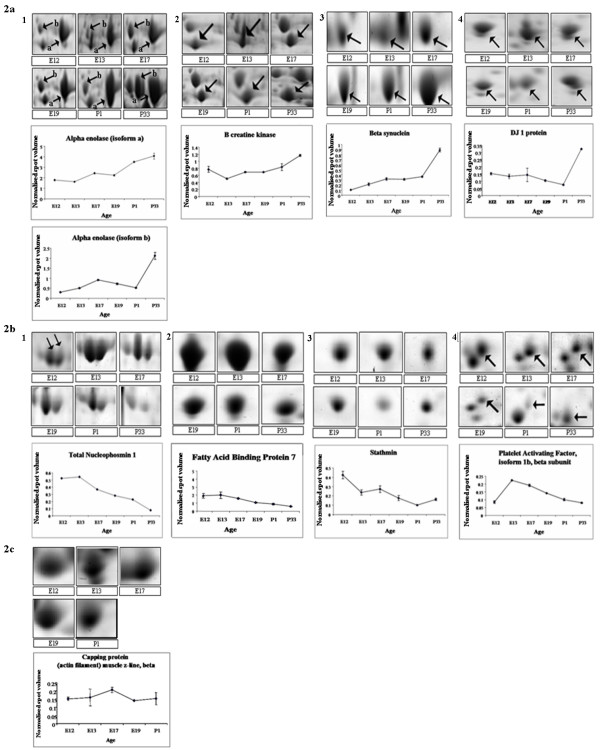
**a Representative 2D montage images showing protein expression changes from E12, E13, E17, E19, P1 and P33 chick retina**. Shown in the gel montage images are proteins which displayed a general increase in expression between E12 and P33 (Adult/A type proteins) together with graphical representation of the change in protein expression. The vertical axis shows spot normalized volume and each point on the line graph represents the average spot abundance expressed as normalized volume ± SEM,*n *= 3). For clarity, where there is more than one spot in an image arrows indicate the spots that were identified by MS: (1) Alpha Enolase (spots 2, 3 and 4 in Table 2), (2) B creatine kinase (spot 6, Table 2), (3) Beta synulcein (spot 8, Table 2) and (4) DJ1 Protein (spot 10, Table 2). b Representative 2D montage images showing protein expression changes from E12, E13, E17, E19, P1 and P33 chick retina. Shown in the gel montage images are proteins which displayed a general decrease in expression between E12 and P33 (Juvenile/J type proteins) together with graphical representation of the change in protein expression. The vertical axis shows spot normalized volume and each point on the line graph represents the average spot abundance expressed as normalized volume ± SEM,*n *= 3) For clarity, where there is more than one spot in an image arrows indicate the spots that were identified by MS: (1) Nucleophosmin 1 (spots 1a & 1b, Table 2), (2). FABP7 (spots 7a & 7b, Table 2), (3) Stathmin 1 (spot 9, Table 2) and (4) Platelet activating factor (spot 5, Table 2). c. Representative 2D montage images showing protein expression changes from E12, E13, E17, E19, P1 and P33 chick retina. Shown in the gel montage image is a protein which displayed a transient change in expression during retinal development together with graphical representation of the change in protein expression. The vertical axis shows spot normalized volume and each point on the line graph represents the average spot abundance expressed as normalized volume ± SEM,*n *= 3): capping protein (spot 12, Table 2).

Progenesis image analysis revealed that the vast majority of protein spots displayed no significant observable change in expression between E12 and P33. However, 234 protein spots exhibited decreased expression ≥ 1.5 fold, and 198 proteins with increased expression ≥ 1.5 fold between E12 and P33, respectively. Proteins identified by MS (Table 2) were classified according to Haniu *et al*. (2006)[29]: (1) Proteins that exhibited significant increased expression during retinogenesis from E12 to P33 (adult/A type) (Fig 2a 1–4); (2) proteins that revealed significant decreased expression during retinogenesis from E12 to P33 (juvenile/J type) (Fig 2b 1–4); (3) proteins that showed transient changes between E12 and P33 (transient/T type) (Fig 2c); and (4) proteins that displayed no significant variation during retinal development (Constitutive/C type). The average normalised volume and fold change in expression for each identified protein was calculated (Table 3).

**Table 2:** Proteins identified by mass spectrometry

**Spot Number**	**Protein**	**Accession Number**	**Source**	**Mowse Score**	**% Sequence Coverage**	**Mr (Da)**	**Calculated pI**	**MS System Used**
1a	Nucleophosmin 1	gi| 45383996	*Gallus gallus*	86	36	32840	4.66	MALDI

1b	Nucleophosmin 1	gi|45383996	*Gallus gallus*	86	36	32840	4.66	MALDI

2	Alpha Enolase	gi| 46048768	*Gallus gallus*	75	5	47275	6.17	ESI-MS/MS

3	Alpha Enolase	gi| 119338	*Anas platyrhynchos*	70	6	47210	6.37	ESI-MS/MS

4	Tau-crystallin/Alpha Enolase)	gi| 21325980	*Crocodylus palustris*	73	5	47480	6.23	ESI-MS/MS

5	Platelet activating factor, isoform Ib, beta subunit	gi|71897151	*Gallus gallus*	128	55	25665	5.78	MALDI

6	Chain A, Crystal Structure Of Chicken Brain-Type Creatine Kinase	gi|6573489	*Gallus gallus*	56	22	42998	5.93	MALDI

7a	FABP7	gi|45384320	*Gallus gallus*	77	16	14917	5.61	ESI-MS/MS

7b	gi|45384320	FABP7	*Gallus gallus*	77	16	14917	5.61	ESI-MS/MS

8	Beta Synuclein	gi|45382773	*Gallus gallus*	574	44.4	14063	4.4	MALDI

9	Stathmin	gi|50053682	*Gallus gallus*	97	47	17072	6.18	MALDI

10	DJ1	gi| 66267682	*Alligator mississippiensis*	114	16	20200	6.33	

11a	Gamma Actin	gi|113277	*Emericella nidulans*	59	7	41622	5.64	ESI-MS/MS

11b	Gamma Actin	gi|113277	*Emericella nidulans*	59	7	41622	5.64	ESI-MS/MS

12	Capping protein actin filament muscle Z line, beta	gi|45382905	*Gallus gallus*	153	68	33110	5.43	MALDI

13	Enhancer of rudimentary homologue	gi|57529979	*Gallus gallus*	90	50	12449	5.63	MALDI

14	tubulin, alpha 2	gi|34740335	*Mus musculus*	128	48	50820	4.94	MALDI

15	Triosphosphate isomerase	gi| 45382061	*Gallus gallus*	153	85	26832	6.71	MALDI

16	Tubulin, beta 2B	gi|52138699	*Gallus gallus*	113	32	50385	4.78	MALDI

17	Tubulin, beta 2B	gi|52138699	*Gallus gallus*	157	54	50377	4.78	MALDI

18	Dimethylarginine dimethylaminohydrolase	gi| 45383392	*Gallus gallus*	111	54	31674	5.44	MALDI

19	PREDICTED: hypothetical protein	gi| 118092623	*Gallus gallus*	113	58	33129	5.53	MALDI

20	PREDICTED: similar to TGF-beta interacting protein 1	gi| 50759828	*Gallus gallus*	126	68	36866	5.38	MALDI

**Table 3:** Protein expression changes during chick retinogenesis: Shown are the fold changes in expression between E12 and P33 along with ANOVA p value

**Protein**	**Fold Change in expression between E12 and P33**	**ANOVA (p value)**
Alpha Enolase isoform a	↑* 2.27	< 0.001

Alpha Enolase isoform b	↑ 6.95	< 0.001

B type creatine kinase	↑ 1.52	< 0.05

Beta synuclein	↑ 8.6	< 0.01

DJ 1	↑ 2.1	< 0.05

Nucleophosmin 1	↓** 5.7	< 0.001

Platelet activating factor	↓ 2.8	< 0.05

FABP7	↓ 3.1	< 0.005

Stathmin	↓ 2.6	< 0.001

*↑ indicates a fold increase

** ↓ indicates a fold decrease

**Figure 3:**
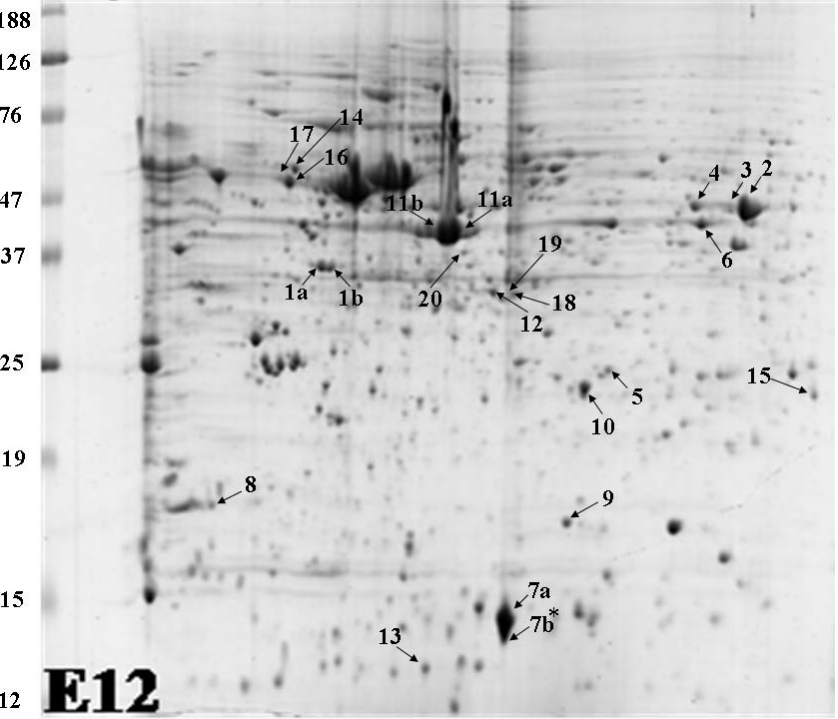
**Chick retinal proteins were subjected to 2D gel electrophoresis, separated through pH 4–7 and stained with Colloidal Coomassie blue**. A representative 2-D gel image with the spots identified by MS analysis is shown. * This spot resolved as two distinct spots at older ages, two spots were identified by MS as FABP7.

Proteins identified by MS included alpha and beta-tubulin, alpha enolase (resolved as three distinct spots), B-creatine kinase, gamma-actin, platelet-activating factor (PAF), PREDICTED: similar to TGF-beta interacting protein 1, capping protein (actin filament muscle Z line), nucleophosmin 1 (NPM1) (resolved as two distinct spots), dimethylarginine dimethylaminohydrolase, triosphoaphate isomerase, DJ1, stathmin, fatty acid binding protein 7 (FABP7/B-FABP) (resolved as two distinct spots at later stages of development), beta-synuclein and enhancer of rudimentary homologue (Table 2 & Fig 3).

## Discussion

Knowledge of proteins expressed during retinogenesis is pivotal to gaining a better understanding of the events that drive retinal neurogenesis and synaptogenesis, both factors are central to the generation of the mature retina and to its maintenance throughout adulthood. This basic information may also provide vital clues to direct the development of stem cell therapies to treat a spectrum of sight threatening degenerative retinal diseases.

This investigation demonstrated the feasibility of employing proteome analysis of the embryonic and post hatch chick retina to generate spatio-temporal maps of protein expression and to identify a number of proteins that were developmentally regulated. 2D PAGE resolved on average 1514 protein spots per gel in the pH range 4–7. Although wide range IPG strips such as pH 3–10 would give a wider pH separation range on a 2D gel, there will be a trade off with resolution. A pH 4–7 strip will only resolve proteins with a pI between 4 and 7, however, it will provide much better spot resolution as it separates the proteins over the same distance as a pH 3–10 strip, proteins may therefore be resolved much more clearly on a narrower pH range than they would be on a wider one.

Identification of proteins was performed using MALDI and ESI MS. In order to make a positive protein identification the matched peptides were always related back to the raw spectra to check the matched peptides were the most intense peaks in the spectra. The search results were then also evaluated by the Mowse score. In Table 2 proteins identified by MALDI all had Mowse scores greater than 79 and were significant (p < 0.05), scores lower than this (proteins identified by ESI MS), although not significant in terms of the Mowse score were still the best matches. Because the Mowse score depends on the number of sequences in the database at the time of the search, species with small database sizes (chicken at that time had a small database size) the Mowse score-based approach was not the most appropriate to determine the significance of a match. In an ideal situation the best match and the most significant match would be the correct match, however, Mowse score significance is highly dependent on the data quality, and, the data generated by ESI MS/MS was not as high a quality as that produced by MALDI, there were less digested fragments and the equipment seemed to be less sensitive, there were therefore less mass values to search the database with. In these cases matches were critically assessed, they were compared to existing 2D gel databases and assessed by correspondence of the experimental pI and molecular weight to the pI and molecular weight based on the spot's position within the gel. These proteins were also compared to proteins that had previously been identified from our lab from porcine retinal 2D gels.

The majority of protein spots on the gels displayed conserved expression densities between the ages studied, however from E12 to P33 approximately 198 protein spots increased ≥ 1.5 fold (p < 0.05), and, 234 decreased ≥ 1.5 fold (p < 0.05). Expression changes were evident in a large population of proteins below the 1.5 fold threshold, but these proteins were not subjected to further analysis in this study as they fell outside the p < 0.05 significance level.

Haniu *et al*. (2006) [29] introduced the concept 'proteomic trajectory mapping', to profile the spatiotemporal kinetics of protein expression in the mouse retina. Likewise, in this study proteins were placed into four groups based upon this basic classification system. Accordingly, proteins that decreased in expression from E12 to P33 (i.e. highly expressed during early retinogenesis) were termed 'juvenile' 'J' type proteins, 4 of the identified proteins belonged to this group. The second group of proteins, 'adult' 'A' type, gradually increased in expression during retinogenesis, 4 of the identified proteins were more highly expressed in the later stages of retinal development. The third group of proteins were termed 'transient' 'T' type and 1 identified protein showed significant changes in expression midway through retinogenesis at some point between E12 and P33, and, finally 7 proteins that showed no detectable significant variation from E12 to P33 were termed 'constitutive' 'C' type.

Mizukami *et al*. (2008) [23] studied chick retina at three embryonic ages and identified two of the proteins also identified in the present study, the differences in proteins identified between this study and that of Mizukami *et al*. (2008) [23] may reflect the fact that a different method of protein extraction was employed in the present study and a narrower pH range (pH 4–7) was used which allows for much sharper resolution of proteins with pIs falling within this pH range.

Nucleophosmin 1 (NPM1) was classified as a J-type protein, two spots on the 2D gels were identified as NPM1, the horizontal resolution along the acidic to basic axis was indicative of some form of as yet unidentified post translational modification (PTM). NPM1 is a member of a family of nuclear chaperone proteins that are implicated in aiding the proper assembly of nucleosomes and maintaining chromatin structure, both key events in preserving cellular function [30,31]. Previous studies have shown that NPM1 knockout causes embryonic lethality between E11.5 and E12.5 [30] and knockout animals displayed many developmental abnormalities including complete absence of eyes and deficient brain organogenesis [32]. The present study demonstrated that NPM1 was highly expressed at early stages of retinogenesis and exhibited a steady decline in expression with retinal maturation. This observation allied with the NPM1 knockout murine data [32] would suggest this protein may play an important role in the early stages of ocular and retinal development.

Fatty Acid Binding Protein 7 (FABP7/B-FABP) was identified and its detection was consistent with previous studies reporting maximal expression at E7/E9 in the chick retina, with a general decrease in expression thereafter [33-36]. The down-regulation of this protein has also been previously reported in postnatal chick retina [21]. FABPs are involved in signal transduction, lipid trafficking toward specific metabolic pathways, and fatty acid transport and metabolism during neuronal development [37-39]. FABPs are reported to be important in the development of the characteristic lipid compositions of neural tissue [40] and studies suggest that FABP expression in embryonic chick retina may be linked to the demand for polyunsaturated fatty acids during retinogenesis and that these molecules may sequester fatty acids in preparation for neurite outgrowth as retinal cells differentiate [40].

Stathmin 1 was classified as a J-type protein and showed decreased expression with retinal development. Previous studies detected elevated expression in the nervous system where it may be involved in the regulation of developmental cell proliferation and differentiation, and plasticity [39,40]. It has also been reported to exhibit high expression at early stages of retinal development which decreased with retinal maturation [29,35].

The present study detected an increase in B-CK expression from ED12 through to P33 (A-type protein), it is plausible that it may be involved in retinal cell proliferation, migration and differentiation. High B-CK levels were previously detected in the chick retina, with intense immunostaining evident in the GCL during early development (E2–E5) and in differentiating photoreceptor cells at E18 [43]. Retinal expression increased until hatching; this was followed by a slight decline with constitutive expression during adulthood [43].

Platelet-activating factor (PAF) acetylhydrolase, isoform 1b, alpha 1 subunit exhibited a slight increase from E12 to E13, then gradually decreased into adulthood and was classified as a J-type protein. It is an ether-linked phospholipid derived from the metabolism of membrane phospholipids [44]. Previous studies detected PAF expression in the chick retina in response to specific neurotransmitter stimuli [45-47]. However, the exact role of PAF remains unclear; it may be involved in intercellular signaling [48] and/or neuronal differentiation [49].

Beta-synuclein exhibited an A-type profile; its expression increased 8.6 fold from E12 to P33. Synucleins are small, soluble proteins primarily expressed in neural tissue [50] and in certain tumours [51]. This family of molecules has been extensively studied due to their involvement, particularly alpha-synuclein, in neurodegenerative pathologies such as Alzheimer's disease [52]. Alpha, beta and gamma synuclein are present in the retina and intense beta-synuclein has been detected in the IPL [50].

DJ1 protein displayed a gradual increase in expression form E12 to P33, and was classified an A-type protein. Haniu *et al*. [29] identified this protein in the mouse retina where it exhibited constitutive expression. This observation may reflect important differences in the ratios of cell types expressed in the developing cone-enriched chick versus the rod-dominant murine retina. DJ-1 has been implicated in the control of cell survival [53] and altering p53 activity [54], it was also found to be highly expressed in neuronal cells and to a lesser extent in non-neuronal cells in the murine brain [55]. Previous studies have reported that DJ-1 plays an antioxidant role by protecting against oxidative damage and in the prevention of apoptosis [56, 57]. Presently there is no reported association between DJ1 and retinal function, although it is likely to be an important retinal protein based on previous reports of its role as an antioxidant and in the prevention of mitochondrial-induced cell death.

Capping protein (actin filament muscle Z line) a T-type protein is reported to control the addition of actin subunits to actin filaments and it also nucleates actin polymerization *in vitro *[56]. The increase in capping protein expression at E17 coincides with the morphogenesis of the photoreceptor outer and inner segments; however, further analysis would be required to substantiate linkage.

Additional proteins, alpha and beta-tubulin, gamma-actin, PREDICTED: similar to TGF-beta interacting protein 1, dimethylarginine dimethylaminohydrolase, triosphoaphate isomerase, enhancer of rudimentary homologue were also identified as constitutive type proteins, i.e. their expression did not significantly change during development.

Analysis of any developmentally driven tissue that exhibits significant changes in cell number with the concomitant acquisition of distinct cellular phenotypes ensures that its study is inherently complex and the data generated must be interpreted with caution. Nonetheless, this study has demonstrated the potential of proteomics to profile protein expression in the developing chick retina and has generated data on a number of developmentally regulated proteins that may be intrinsic to retinal development and maturation. In order to begin to understand and treat such debilitating diseases as RP we must first unravel the events driving normal retinogenesis. The identification of proteins expressed during retinogenesis and the analysis of their expression levels throughout retinal maturation will only increase our knowledge of the subject, and, with the possibility of stem cell therapy as a means to treat RP, knowledge of protein expression during critical stages of retinal development raises the possibility of directing cell type differentiation and integration by manipulating trophic factors and protein levels to minimize rejection. The continued generation of substantive mass spectrometric data for additional protein populations that exhibit fold changes during development is an absolute necessity and will enable the establishment of a comprehensive retinal development proteome database. With the establishment of such databases we can then begin to construct a clearer picture of such processes as retinogenesis which may ultimately increase our understanding of such complex developmental mechanisms.

## Competing interests

The authors declare that they have no competing interests.

## Authors' contributions

The work was funded by the Department for Employment and Learning and Fight for Sight, UK. SF conceived the study, harvested retinae, performed 2DE, gel image analysis and prepared the manuscript. JLR performed some of the MS analysis and database searches. MW provided the animals. AH performed some of the MS analysis. WJC helped draft the manuscript and was an academic supervisor. AWS was an academic supervisor.
